# Disparities in Receipt of Radiotherapy and Survival by Age, Sex, and Ethnicity among Patient with Stage I Follicular Lymphoma

**DOI:** 10.3389/fonc.2016.00101

**Published:** 2016-04-28

**Authors:** Amir Bista, Sandhya Sharma, Binay Kumar Shah

**Affiliations:** ^1^Guthrie Robert Packer Hospital, Sayre, PA, USA; ^2^PeaceHealth United General Hospital, Sedro Woolley, WA, USA

**Keywords:** follicular lymphoma, radiotherapy, survival, SEER, ethnicity

## Abstract

**Background:**

Radiotherapy (RT) is a first-line treatment option for stage I follicular lymphoma (FL). We studied disparities in receipt of RT and survival among patients with stage I FL.

**Methods:**

Adult patients (age ≥18 years) with stage I FL, as the first primary cancer, diagnosed between 1992 and 2007 were identified using Surveillance, Epidemiology, and End Results (SEER) 18 database. Study population was divided into various subgroups based on age, sex, race, and marital status. Factors associated with receipt of RT and survival, among patients receiving RT, was evaluated using regression analysis and Cox PH modeling, respectively. SEER*Stat was used to compute 1- and 5-year RS for various subgroups and compared using *Z* score.

**Results:**

Of the total 7315 patients (median age: 64 years), 2671 (36.5%) received RT. African-Americans, older age group, and single and separated/divorced/widow marital status predicted omission of RT. The 1- and 5-year RS were significantly better in patients receiving RT. In multivariate analysis, male sex, age <60 years, Caucasian race, and married marital status were found to be independent predictor of better RS among patients receiving RT (*P* < 0.0001).

**Conclusion:**

This study showed that 36.5% patients with stage I FL received RT. Survival rates were significantly better for patients who received RT.

## Introduction

Follicular lymphoma (FL) is the second most common non-Hodgkin’s lymphoma (NHL) with incidence of 3.7 per 100,000 in the United States (1). In a recent analysis of Surveillance, Epidemiology, and End Results (SEER) database, 26% of patients with FL had stage I disease (2). Radiation therapy is an effective therapy for limited stage FL and may be curative in significant proportion of the patients (3–5). In fact, National Comprehensive Cancer Network recommends radiotherapy (RT) as the preferred option. A retrospective analysis of SEER database for stage I FL diagnosed from 1973 to 2004 showed that upfront RT significantly improved disease specific and overall survival of patients (6). Another population-based study showed significant improvement in survival of patients with RT compared to those who did not receive RT (5- and 10-year OS rates of 86 and 68%, respectively, compared with 74 and 54%, respectively, with *P* < 0.0001) (7).

We conducted this population-based study to analyze factors associated with the receipt of RT and survival among patients with stage I FL.

## Methods and Methodology

Surveillance, Epidemiology, and End Results 18 registry 2014 submission was used to identify adult patients with stage I FL as first primary. SEER database is the National Cancer Institute sponsored population-based database that collects incidence and survival data on cancer cases from various locations and sources in the United States. SEER 18 covers about 27.8% of total US population (8). SEER program maintains high case ascertainment of about 98% by conducting rigorous quality control studies every other year (9).

Lymphoma subtype recode/WHO 2008 was used to identify patients with FL and AJCC lymphoma Ann Arbor Stage (1983+) was used to identify patients with stage I FL. Adults patients with age 18 years and above with stage I FL as the first primary cancer diagnosed from 1992 to 2007 were included in the study. Patients who were microscopically confirmed and were included in research database were included in the study, while patients with unknown age, unknown year of diagnosis, incomplete demographic date, alive with no survival time, no data on radiation therapy, death certificate only, and autopsy only cases were excluded from the study. Patients who received RT other than beam therapy (including radioactive implants, radioisotopes, combination of beam with implants or isotopes, unknown type method or source of radiation) were also excluded.

The study population was divided into various subgroups based on age (<60 and ≥60 years), sex, ethnicity [Caucasians (C), African-Americans (AAs), and Others (O)], and marital status [married (M), single (S), and separated/divorced/widowed (S/D/W)]. Binomial logistic regression was used to compute unadjusted and adjusted odds ratio (OR) to investigate on factors associated with receipt of RT, using SPSS version 20. SEER*Stat version 8.2.1 was used to compute 1- and 5-year relative survival (RS) for patients in no RT group and RT group, among various subgroups, and compared using *Z* score. Two-sided *P*-values were computed from the *Z* score for test of significance; *P* value <0.05 was considered to be statistically significant. RS is defined as the ratio of the observed survival of a group of cancer patients to survival of cancer free population in specified time period and specified place (10). Since the survival of cancer free population is hard to estimate, the survival of the whole population is taken for the calculation with assumption that death of cancer patient is negligible compared to general population (11). The survival curves for RT and no RT groups were plotted using RS obtained with life table using SEER*Stat and actuarial method with 1-month intervals. Cansurv Software and Cox proportional hazard model were used to compare 5-year RS among RT and no RT group, after adjusting for age, sex, race, and marital status, and to compare factors associated with 5-year RS among patients receiving RT.

## Results

### Study Population

**Figure 1:**
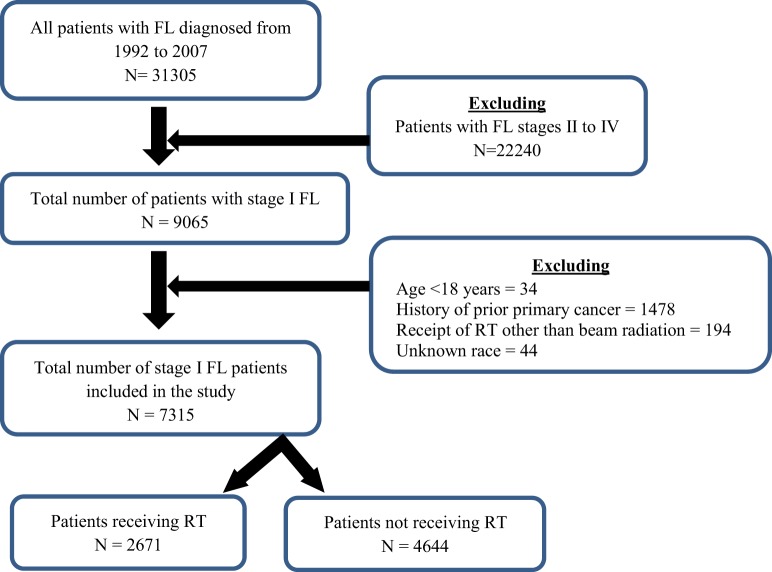
**Consolidated Standards of Reporting Trials (CONSORT) diagram**.

A total of 7315 patients, with Ann Arbor stage I, were included in the study (Figure 1). The median age at diagnosis was 64 years with range of 18–98 years. Majority of the patients were Caucasians (91.1%), ≥60 years (59.2%), females (50.8%), and married (64.6%). Patients’ demographics are available in Table 1.

**Table 1:** **Demographic distribution of patients receiving RT and not receiving RT**.

Characteristics	All	No radiotherapy (*n* **=** 4644)	Radiotherapy (*n* **=** 2671)	*P* value*
Sex				0.039
Male	3600 (49.2%)	2243 (48.3%)	1357 (50.8%)	
Female	3715 (50.8%)	2401 (51.7%)	1314 (49.2%)	
Age				<0.001
<60 years	2981 (40.8%)	1708 (36.8%)	1273 (47.7%)	
≥60 years	4334 (59.2%)	2936 (63.2%)	1398 (52.3%)	
Race				
Caucasians	6662 (91.1%)	4218 (90.8%)	2444 (91.5%)	<0.001
AA	308 (4.2%)	225 (4.8%)	83 (3.1%)	
Others	345 (4.7%)	201 (4.3%)	144 (5.4%)	
Marital status				<0.001
Married	4722 (64.6%)	2893 (62.3%)	1829 (68.5%)	
Single	776 (10.6%)	498 (10.7%)	278 (10.4%)	
S/D/W	1481 (20.2%)	999 (21.5%)	482 (18.0%)	
Unknown	336 (4.6%)	254 (5.5%)	82 (3.1%)	
Total	*N* = 7315	4644 (63.5%)	2671 (36.5%)	

*P value by chi square.

*AA, African-Americans; S/D/W, separated/divorced/widowed*.

Among the patients with stage I FL, 36.5% patients received RT. The demographics of the patients with receipt of RT can be found in Table 1.

### Factors Associated with Receipt of RT

Radiotherapy was omitted more often in elderly patients compared to younger patients (unadjusted OR of 0.64, *P* < 0.001; adjusted OR of 0.64, *P* < 0.001) and in AAs compared to Caucasian (unadjusted OR of 0.64, *P* < 0.001; adjusted OR of 0.61, *P* < 0.001) patients. There was no significant difference in the receipt of RT for “Others” race compared to Caucasian (unadjusted OR of 1.24, *P* 0.06; adjusted OR of 1.19, *P* 0.14). There was no gender difference in the receipt of RT, when adjusted for other variables. When adjusted for other variables, there was no significant difference in receipt of RT for “Others” race compared to Caucasians (adjusted OR 1.19, P 0.14). The probability of receiving RT decreased with recent year of diagnosis (unadjusted and adjusted OR of 0.97 and 0.98 for 1-year increase in year of diagnosis with *P* < 0.001 for both). The details of the factors associated with RT can be found in Table 2.

**Table 2:** **Factors associated with receipt of RT in patients with stage I follicular cancer**.

Parameters	Unadjusted OR	95% CI	*P* value	Adjusted OR	95% CI	*P* value
Age	0.639	0.580–0.704	<0.001	0.637	0.577–0.704	<0.001
Sex	0.905	0.822–0.995	0.039	0.958	0.867–1.058	0.394
Race						
Caucasian	Reference	Reference				
AA	0.637	0.493–0.823	0.001	0.606	0.467–0.785	<0.001
Others	1.236	0.993–1.540	0.058	1.179	0.944–1.473	0.146
Marital status						
Married	Reference	Reference				
Single	0.883	0.754–1.034	0.123	0.835	0.711–0.981	0.028
S/D/W	0.763	0.675–0.863	<0.001	0.858	0.753–0.977	0.021
Unknown	0.511	0.395–0.659	<0.001	0.526	0.406–0.681	<0.001
YOD	0.973	0.962–0.984	<0.001	0.975	0.964–0.986	<0.001

*CI, confidence interval; age, ≥60 vs. <60 years; sex, female vs. male; AA, African-Americans; S/D/W, separated/divorced/widowed; YOD, year of diagnosis*.

### Comparison of Survival among Patients in RT and No RT Group

Patients receiving RT had significantly better 1- and 5-year RS when compared to those who did not receive RT (1-year RS: 99.9 ± 0.3 vs. 96.0 ± 0.4%, *P* < 0.0001, and 5-year RS: 97.3 ± 0.7 vs. 88.4 ± 0.7%, *P* < 0.0001). When the RS obtained by life table and actuarial method with interval of 1 month was plotted for patients in RT and no RT group, the curves diverged early in the course and the difference increased with time. On subgroup analysis, 1-year survival was significantly better in RT group compared to no RT group for all cohorts except AAs, “Others” race, and younger patients (<60 years). There was no improvement in 5-year survival for patients of “Others” race in RT group compared to no RT group.

### Factors Associated with Survival: Cox PH Model

On Cox PH modeling, receipt of RT was independent predictor of better 5-year survival after adjusting for age, sex, race, and marital status (HR of 0.81 with *P* value of <0.0001).

Among patients receiving RT, female sex (HR of 0.088, *P* < 0.0001), older age (HR of 0.33, *P* < 0.0001), and Caucasian race (HR of 11.22 and 8.27 for AA vs. Caucasian and Others vs. Caucasian, respectively, with *P* < 0.0001) were associated with better 5-year survival compared to their counterparts, after adjusting for other covariates. Also, married patients had better survival compared to single or S/D/W (Table 3).

**Table 3:** **Factors associated with survival among stage I FL cases receiving RT**.

Parameters	Estimate	Hazard ratio	*P* value
Sex (female vs. male)	2.4323	0.0878	<0.0001
Age (≥60 vs. <60 years)	1.1073	0.3305	<0.0001
Race			
Caucasians		Reference	
African-American	−2.4173	11.2155	<0.0001
Others	−2.1123	8.2672	<0.0001
Marital status			
Married		Reference	
Single	−0.5703	1.7688	<0.0001
S/D/W	−0.1182	1.1254	<0.0001
Unknown	−2.3245	10.2216	<0.0001

*S/D/W, separated/divorced/widowed*.

## Discussion

Current recommendations for treatment of stage I FL include watchful waiting, RT, and combined modality therapy (CMT). To the best of our knowledge, the recommendations are not based on prospective randomized controlled trials comparing different treatment modalities. A retrospective study by Advani et al. found survival for watchful waiting comparable to those who received immediate intervention, with 5-year overall survival for the whole population of 97% (12). There is a need for more research to identify factors or biomarkers that may help us identify individuals who may do well without therapy. Our study showed that less than half (36.5%) of the patients with stage I FL received RT and are consistent with the findings of other studies. In National LymphoCare Study, 56 out of 206 (27%) patients were treated with RT (13). A recent retrospective study utilizing National Cancer Data Base showed that only 21% patients with stage I FL received RT (7). It revealed that the use of RT was independently associated with improved overall survival (5- and 10-year OS 86 and 68%, respectively, compared to 74 and 54% for those who did not receive RT, *P* < 0.0001). Although unclear, several factors, such as physician preference, large abdominal radiation field required, advanced age, concern for side effect, or patient refusal, may be responsible for the omission of RT as initial therapy.

The use of RT was found to be gradually but significantly declining with recent year of diagnosis. Similar to our findings, Vargo et al. found gradual decrease in the utilization of RT in patients with early-stage FL with recent year of diagnosis. It decreased from 37% in 1999 to 24% in 2012 and corresponds with gradual rise in adoption of single agent chemotherapy or watchful waiting for management of these patients (7). Interestingly, a retrospective study using SEER database by Shah et al. showed that the receipt of RT among patients with stage I diffuse large B cell lymphoma gradually declining with recent year of diagnosis (14). Reasons behind decline in the use of RT in lymphoma are not well studied, but advent of newer therapies, including immunotherapy and targeted therapy, may be contributing factors.

We identified demographic factors, such as AA race, singles and S/D/W martial status, and older age to be associated with decreased use of RT. Racial disparities in the receipt of definitive cancer therapy have been shown in multiple studies (15–18). In general, patients from racial or ethnic minorities, mostly AAs, are less likely to receive definitive cancer-directed therapy. Several factors, including lower socioeconomic status, lack of insurance coverage, perceptions and belief of patients, and bias of treating physician, have been attributed to ethnic disparities in cancer treatment (19). Older patients were less likely to receive RT compared to younger patients in our study. Older patients have poor performance status and higher number of comorbidities compared to younger patients (20, 21). Also, even though there has been a demonstration that elderly patients have the same incidence of side effects related to radiation therapy when the stage, performance status, and other clinical status are matched with that of younger patients, physicians are biased when they have to decide on RT in elderly patients (22–24). In one study, physician bias for the elderly patients resulted in significantly lower definitive therapy in elderly patients compared to younger patients with similar comorbidities (25). Lack of support system may contribute to lower utilization of RT among singles and S/D/W patients. Further research is warranted that is aimed at bridging disparities in treatment of these patients.

Our study showed superior survival rates for females. Study by Keegan et al. (26) showed better survival rates for females with lymphoma. Although unclear, other factors, such as differences in biology of lymphoma and differences in metabolism of chemotherapy, may be responsible for the differences in outcomes in these patients.

This study has innate limitations characteristic of population-based registries. The details of the definitive treatment, including dose of radiation, number of cycles of treatment received, receipt of chemotherapy, and/or immunotherapy, such as rituximab, treatment complications, details of prognostic features, including LDH level and performance status, reason for omission of a therapy either from physician or patient prospective, are not known. Strength of this study includes large population size, high-quality data, and long-term follow-up of patients.

## Conclusion

Our study showed that approximately one-third of patients with stage I FL received RT. Elderly patients, AA race, and single or S/D/W as marital status were significant predictor of omission of RT in patients with stage I FL. Among patients receiving RT, old age, female sex, Caucasians, and married marital status predicted better 5-year RS.

## Author Contributions

BS: concept and hypothesis; BS, AB, and SS: collected data; analyzed data, and wrote the manuscript. All authors reviewed and decided to submit the manuscript for publication.

## Conflict of Interest Statement

The authors declare that the research was conducted in the absence of any commercial or financial relationships that could be construed as a potential conflict of interest.
